# Comparative analysis of the water-based nanofluids in semi-circular heat transfer systems for thermohydraulic performance evaluation

**DOI:** 10.1039/d6na00038j

**Published:** 2026-05-18

**Authors:** Ussama Ali, Isam Janajreh

**Affiliations:** a Mechanical and Nuclear Engineering Department, Khalifa University of Science and Technology Abu Dhabi 127788 United Arab Emirates isam.janajreh@ku.ac.ae; b Center for Membrane and Advanced Water Technology, Khalifa University of Science and Technology Abu Dhabi 127788 United Arab Emirates; c Mechanical Engineering Department, University of Engineering and Technology Lahore 54890 Pakistan

## Abstract

This study presents a comprehensive numerical investigation of the thermohydraulic behavior of seven water-based nanofluids, Al, Al_2_O_3_, Cu, CuO, graphene (G), graphene oxide (GO), and hexagonal boron nitride (hBN), within a two-dimensional semi-circular cavity under a transitional regime. Using the transition SST *K–ω* model in the ANSYS Fluent software, simulations were conducted at a Reynolds number of 2500, incorporating nanoparticles at concentrations ranging from 0.5% to 5% by volume. The thermophysical properties were modeled using the established correlations. Key performance indicators included the convective heat transfer coefficient, Nusselt number, cavity wall temperature, thermal effectiveness, pressure drop, pumping power, and thermal performance factor (TPF). Results revealed that G/water and hBN/water nanofluids consistently outperformed others, delivering up to 53% and 42% heat transfer enhancement, respectively, with minimal hydraulic penalties. In contrast, Cu/water and CuO/water nanofluids, while thermally effective, exhibited a significant pressure drop and energy cost. Al/water, Al_2_O_3_/water, and GO/water demonstrated limited thermal gains relative to their pumping demands. These results highlight the importance of balancing the thermal conductivity, dispersion stability, and hydrodynamic performance in the nanofluid design, particularly for applications in electronics cooling and compact heat exchange systems.

## Abstract 2

Graphene and hBN water-based nanofluids exhibit the highest thermohydraulic performance in a semi-circular cavity, achieving up to 53% heat-transfer enhancement with a moderate pressure drop compared with the Al, Al_2_O_3_, Cu, and CuO nanofluids.

## Graphical abstract

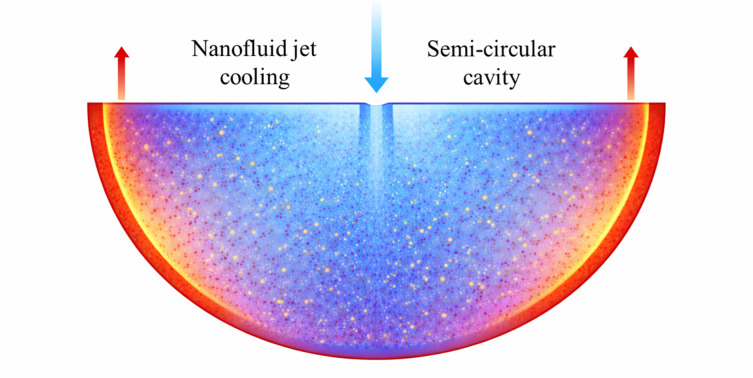

## 1. Introduction

In recent decades, the pursuit of enhanced thermal performance in engineering systems has gained extensive research attention in advanced heat transfer fluids. Among these, nanofluids, which are colloidal suspensions of nanoparticles within conventional base fluids such as water, ethylene glycol, or oil, have emerged as a groundbreaking innovation.^1,2^ First introduced by Choi and Eastman in 1995,^3,4^ nanofluids exhibit significantly improved thermophysical properties compared with their base fluids, primarily due to the high thermal conductivity and large surface area of their dispersed nanoparticles.^5,6^ These enhancements lead to greater convective heat transfer coefficients, improved thermal conductivity, and better energy efficiency, especially in compact and high-flux thermal systems.^7,8^ Furthermore, nanofluids offer potential advantages such as reduced size and weight of heat exchangers^9,10^ and the capability to tailor fluid properties by selecting appropriate nanoparticle types, sizes, and concentrations. Therefore, nanofluids are now considered highly suitable for a wide array of applications, including electronic cooling,^11,12^ fabrication of automotive radiators,^13^ solar thermal collection,^14^ and advanced manufacturing processes.^15^

Among the most widely researched oxide-based nanofluids, aluminum oxide (Al_2_O_3_) nanofluids have consistently demonstrated notable thermal performance enhancements in various engineering systems.^16–18^ The high surface area, thermal conductivity, and chemical stability of Al_2_O_3_ nanoparticles make them an effective choice for augmenting convective heat transfer. Vajjha *et al.* (2010)^19^ conducted a numerical investigation to assess the thermal performance of Al_2_O_3_ and CuO nanofluids in an ethylene glycol–water mixture under laminar flow conditions through flat tubes of an automotive radiator. Their findings showed a notable improvement in the convective heat transfer coefficient when using nanofluids compared with the base fluid. Specifically, the Al_2_O_3_ nanofluid at a volume concentration of 10% exhibited an average heat transfer coefficient approximately 91% higher than that of the base fluid. Additionally, the study found that both the local and average friction factors, as well as the convective heat transfer coefficients, increased with the nanoparticle concentration. Similarly, Elsebay *et al.*^20^ conducted a numerical study to investigate the impact of Al_2_O_3_ and CuO nanoparticles on the thermal and hydraulic performance of nanofluids flowing through the flat tubes of an automobile radiator. Their findings revealed that both nanofluids significantly enhanced the average heat transfer coefficient by up to 45% for Al_2_O_3_ and 38% for CuO compared with pure water. However, the improvements came at the cost of increased friction factor and pressure drop, particularly at higher nanoparticle concentrations. A resizing analysis further showed that the radiator tube length could be reduced by 11.7% for Al_2_O_3_ and 9.8% for CuO, maintaining the same cooling performance. Despite the benefit of more compact radiator design, the study noted a substantial increase in pumping power (up to 80%), emphasizing the trade-off between thermal enhancement and hydraulic efficiency. Similarly, Andreozzi *et al.*^21^ explored Al_2_O_3_/water nanofluids in ribbed channels under turbulent conditions and noted that the thermal performance improved consistently with the increase in particle concentration, though at the cost of higher pressure losses.

In a numerical study by Lima-Téllez *et al.*,^22^ an Al_2_O_3_/water nanofluid was employed in a photovoltaic (PV) cooling channel equipped with baffles. The authors reported that a 10% volume concentration of Al_2_O_3_ nanofluid reduced the operating temperature of the PV panel by up to 15%, while increasing the electrical efficiency by approximately 4% at low Reynolds numbers (Re). In a separate numerical investigation using the lattice Boltzmann method, Mohebbi *et al.*^23^ found that although Al_2_O_3_ nanofluids showed slightly inferior performance than CuO nanofluids in heat transfer effectiveness, they still yielded significant Nusselt number improvements, especially at higher Reynolds numbers and particle concentrations. Furthermore, Alshukri *et al.*^24^ numerically assessed turbulent flow in baffled channels and confirmed that Al_2_O_3_ nanofluids at 4% volume fraction offered substantial thermal performance gains while maintaining acceptable hydraulic losses, reinforcing their suitability for high-Reynolds applications. Although aluminum (Al) nanoparticles are generally less stable in water than aluminum oxide (Al_2_O_3_) due to their higher reactivity and tendency to oxidize, they have nonetheless been investigated in studies for their high thermal conductivity and potential to enhance convective heat transfer.^25^ Jamal-Abad *et al.*^26^ conducted an experimental investigation on Al/water nanofluids in spiral coils and found that the thermal conductivity increased by up to 22% at a volume concentration of 2.23%, accompanied by a notable rise in the Nusselt number. Interestingly, although the friction factor increased slightly with the nanoparticle concentration, it remained close to that of the base fluid, implying a negligible pressure drop penalty for Al/water nanofluids.

Copper (Cu)-based nanofluids have demonstrated superior thermal performance compared to other metallic nanofluids due to copper's high intrinsic thermal conductivity.^27–29^ In the study by Jamal-Abad *et al.*,^26^ Cu/water nanofluids outperformed Al/water counterparts, offering up to 26% enhancement in thermal conductivity at 2.23% volume concentration. While the friction factor did increase slightly with the nanoparticle concentration, it remained within practical limits for most engineering systems. Additionally, Nouari *et al.*^30^ conducted a numerical study of Cu/water nanofluid jet cooling in an inverted T-shaped channel and found that increasing the nanoparticle concentration significantly improved the Nusselt number across a wide range of Reynolds numbers. Their findings further confirmed that Cu/water nanofluids offer efficient thermal management solutions in compact electronic cooling systems, where space and thermal loading are both constrained.

Copper oxide (CuO) nanofluids are more commonly studied than those containing metallic copper, primarily due to their greater colloidal stability in aqueous suspensions and superior cost-effectiveness compared to pure copper nanoparticles.^31^ Numerous studies have confirmed the superior performance of CuO/water nanofluids in both laminar and turbulent heat transfer applications. Mohebbi *et al.*^23^ conducted a comparative numerical study using the lattice Boltzmann method in a channel with extended surfaces, where CuO/water nanofluids outperformed Al_2_O_3_- and TiO_2_-based alternatives in terms of heat transfer augmentation. Their results showed that increasing the nanoparticle concentration from 0% to 5% led to a significant increase in the average Nusselt number, especially at higher Reynolds numbers, confirming the strong temperature-dependent performance of CuO nanofluids. Furthermore, Karami *et al.*^32^ conducted a numerical investigation into the turbulent flow of CuO/water nanofluids in grooved cylindrical channels, with particular focus on how the nanoparticle concentration affects thermal performance. Their results confirmed that increasing the CuO volume fraction from 0% to 4% consistently improved the average Nusselt number across all tested geometries and Reynolds numbers, indicating enhanced convective heat transfer. The study also noted that higher nanoparticle concentrations led to increased fluid viscosity and density, which in turn increased the friction factor and pressure drop, especially in grooved channels. Similarly, Lima-Téllez *et al.*^22^ numerically investigated CuO nanofluids in a photovoltaic panel cooling system with baffles. Their findings showed that CuO-based nanofluids yielded a high thermal efficiency improvement, up to 5.67% over water, at low Reynolds numbers, reinforcing the suitability of CuO nanofluids in low-flow-rate thermal applications. In turbulent flow regimes, Alshukri *et al.*^24^ examined CuO/water nanofluids flowing through baffled rectangular channels and observed a 10.3% improvement in the heat transfer coefficient compared to water alone, with a thermal performance factor approaching 2 at Re = 5000.

Besides the traditional Al- and Cu-based nanofluids, graphene-based nanofluids have garnered increasing attention due to graphene's extraordinary thermal conductivity, high aspect ratio, and surface area, making them especially suitable for high-performance cooling applications.^33–35^ Almutter *et al.*^36^ used molecular dynamics simulations to study the impact of graphene nanoparticle size on Brownian displacement and thermophoretic behavior in water-based graphene nanofluids. They reported that increasing the nanoparticle size from 0.5 nm to 1 nm led to enhanced Brownian motion and thermophoresis, which translated to a substantial rise in thermal conductivity from 0.36 to 0.51 W m^−1^ K^−1^, reinforcing the efficiency of larger graphene nanoplatelets in promoting convective heat transfer. Arzani *et al.*^37^ performed both experimental and numerical analyses to explore how various parameters affect the thermal behavior of water-based graphene nanoplatelet (GNP) nanofluids flowing through an annular channel. Their results revealed that a GNP concentration of 0.1% by weight (wt%), among 0% to 0.1%, yielded the greatest improvement in the convective heat transfer coefficient, showing a 22% increase compared to pure water. However, they also noted that higher nanoparticle concentrations led to a rise in the friction factor, which in turn caused a noticeable increase in pressure loss throughout the system. Similarly, AbdRabbuh *et al.*^38^ performed both experimental and CFD studies on GNP nanofluids in aqueous solutions functionalized using gallic acid. They tested the thermal performance of an annular passage with various nanoparticle concentrations. The nanofluids exhibited an enhancement in thermal conductivity of up to 18.25%, accompanied by a 12.11% increase in the convective heat transfer coefficient at a concentration of 0.1 wt%. The pressure drop also increased with the nanoparticle concentration, but the performance index was highest at 0.025 wt%, indicating the best balance between thermal gain and pumping cost.

Graphene oxide (GO), a derivative of graphene, offers enhanced dispersibility in water due to its oxygen-containing functional groups, and has been widely adopted in nanofluid applications demanding stable suspensions.^39–41^ Bai *et al.*^42^ examined the convective heat transfer performance and stability of graphene oxide/water nanofluids in a simulated spacecraft fluid loop. Their results showed that the nanofluids exhibited enhanced convective heat transfer compared to the base fluid, particularly in the 298–358 K temperature range, where the structure of the GO remained stable. The Nusselt number of the nanofluid was up to 43% higher than that of water at low heating powers. In another study, Yusuf *et al.*^43^ examined GO nanofluids in solar collectors using ionic liquid and water mixtures. Their findings showed that GO/water nanofluids exhibited significant thermal enhancement, but the use of ionic liquids further amplified the energy efficiency, achieving up to 37.4% improvement over water.

Hexagonal boron nitride (hBN), often referred to as “white graphene” due to its structural similarity with graphene, has recently emerged as a promising nanomaterial for enhancing the thermal performance in heat transfer systems^44–46.^ With properties such as high thermal conductivity, excellent chemical stability, and electrical insulation, hBN has proven to be an effective additive in water-based nanofluids.^47–49^ Büyükalaca *et al.*^50^ conducted numerical investigations into the use of hBN/water nanofluids in photovoltaic thermal (PVT) collectors and found that at 0.18% volume fraction, thermal efficiency improved significantly, while the electrical efficiency continued to increase with the increase in hBN concentration. hBN/water is marginally better than the graphene/water nanofluids with regard to both thermal and energy efficiencies. Similarly, Rizwan *et al.*^51^ experimentally evaluated a gravitational water vortex heat exchanger using hBN/water nanofluids and observed a maximum heat transfer rate increase from 8490 W to 9998 W, highlighting the strong potential of hBN nanofluids in industrial heat exchange applications. Furthermore, Sofiah *et al.*^52^ applied a response surface method (RSM) to optimize the performance of a PVT system using hBN/water nanofluids, reporting an electrical efficiency of 7.43% and a thermal efficiency of 73.82% under optimal flow and irradiance conditions.

Despite the growing body of research on nanofluids, comparative studies that systematically evaluate the thermal and hydrodynamic behaviors of multiple nanofluids under identical conditions remain limited. The present study aims to fill this gap by offering a comprehensive comparative assessment of seven distinct water-based nanofluids: Al, Al_2_O_3_, Cu, CuO, graphene (G), graphene oxide (GO), and hexagonal boron nitride (hBN), within a controlled semi-circular cavity geometry. This approach allows for a direct and meaningful comparison of their performance in terms of heat transfer enhancement and flow characteristics. Table 1 provides an overview of the water stability and heat transfer potential of the selected nanoparticles used in this work. Stability in water is influenced by factors such as chemical reactivity and surface functionalization, while thermal conductivity and dispersion quality determine the overall heat transfer potential of each nanofluid. The semi-circular cavity, used as the computational domain, closely resembles configurations found in electronics cooling, microchannel heat sinks, and curved duct flows in compact energy systems. Its smooth geometry and potential for uniform heat distribution make it suitable for emerging applications in the thermal management of electronic devices and solar thermal collectors. The rest of the paper is organized as follows: Section 2 outlines the computational methodology used in this study, including turbulence modeling and boundary conditions. Section 3 presents and discusses the results, focusing on key performance metrics such as heat transfer coefficient, cavity wall temperature, Nusselt number, pumping power, and thermal performance factor. Finally, Section 4 provides the main conclusions drawn from this study.

**Table 1:** Summary of the stabilities and thermal characteristics of the nanoparticles used in this study

Nanoparticle	Stability in water	Heat transfer potential
Aluminum (Al)	Poor stability due to high reactivity. Prone to oxidation and agglomeration^53^	High thermal conductivity, but poor dispersion limits practical usage despite strong heat transfer potential^54^
Aluminum oxide (Al_2_O_3_)	Very stable, chemically inert and well-dispersed. Widely used in nanofluids^55^	Moderate conductivity. Widely used due to its balance of thermal performance and excellent stability^56^
Copper (Cu)	Poor stability, oxidizes and sediments quickly without surfactants^57^	Extremely high conductivity, but poor dispersion makes it unstable for long-term applications^58^
Copper oxide (CuO)	More stable than Cu. Good dispersion in water^59^	Lower conductivity, but offers a good balance between thermal performance and stability^60^
Graphene (G)	Low stability, prone to agglomeration unless stabilized with surfactants or functionalization^61^	Very high theoretical conductivity. Excellent heat transfer potential but difficult to stabilize^62^
Graphene oxide (GO)	Good stability due to oxygen functional groups which enhance its water compatibility^63^	Lower conductivity, but more practical due to higher stability compared with graphene^64^
Hexagonal boron nitride (hBN)	Chemically stable in water, but may require surfactants or functionalization to disperse well^65^	High thermal conductivity. Good potential, although cost and dispersion can be limiting factors^66^

## 2. Methodology

### 2.1. Governing equations

The present study solves the steady-state, incompressible, single-phase transitional flow of nanofluids in a two-dimensional (2D) semi-circular cavity using ANSYS Fluent. The governing equations include mass, momentum, and energy conservation, along with turbulence modeling *via* the transition SST *K–ω* model. Nanofluids are assumed to be Newtonian, with the thermophysical properties calculated based on the nanoparticle concentration. The governing equations are expressed in Cartesian coordinates (*x*, *y*). For incompressible, steady-state flow, the continuity equation is as follows:1
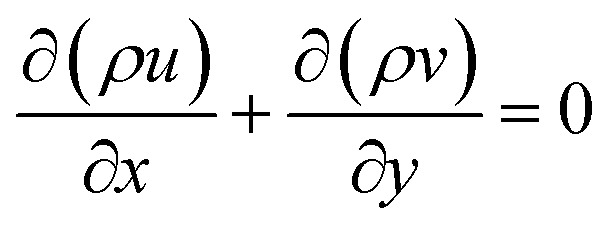
where *ρ* is the fluid density, and *u* and *v* are the velocity components in the *x*- and *y*-directions, respectively. The momentum equations in the *x*- and *y*-directions are given as follows:2a
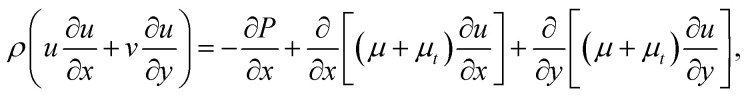
2b
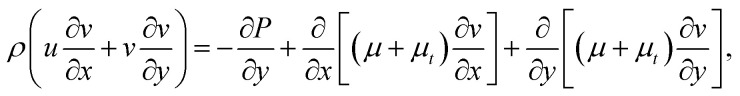
where *P* is the static pressure, *µ* is the dynamic viscosity, and *µ*_*t*_ is the turbulent viscosity. The steady-state energy equation is given as follows:3
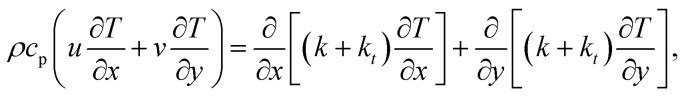
where *T* is the temperature, *c*_p_ is the specific heat capacity, *k* is the thermal conductivity, and *k*_*t*_ is the turbulent thermal conductivity. The transport equations for the turbulent kinetic energy (*K*) and the specific dissipation rate (*ω*) are as follows:4a
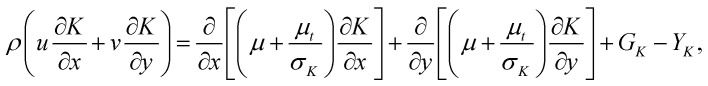
4b
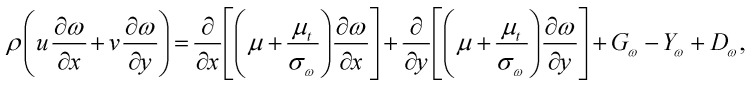
where *G*_*K*_ and *G*_*ω*_ are the generation terms, *Y*_*K*_ and *Y*_*ω*_ are the dissipation terms, *D*_*ω*_ accounts for cross-diffusion, and *σ*_*k*_ and *σ*_*ω*_ are the turbulent Prandtl numbers for *K* and *ω* equations, respectively.

### 2.2 Thermophysical properties

In this study, nanofluids are modeled as single-phase homogeneous mixtures, with their effective thermophysical properties computed based on the established empirical correlations. The base fluid is water, and the nanoparticles used include aluminum (Al), aluminum oxide (Al_2_O_3_), copper (Cu), copper oxide (CuO), graphene (G), graphene oxide (GO), and hexagonal boron nitride (hBN), all with a mean particle diameter of 10 nm. Nanoparticle volume fractions (*ϕ*) considered were 0.5%, 1%, 3%, and 5%. The following equations were used to calculate the nanofluid properties, based on Corcione *et al.*^67^

Density of the Nanofluid:

5 *ρ*_nf_ = *ϕρ*_p_ + (1 − *ϕ*)*ρ*_bf_,5

where *ρ*_nf_ is the density of the nanofluid, *ρ*_p_ is the density of the nanoparticle, and *ρ*_bf_ is the density of the base fluid (water). The term *ϕ* represents the volume fraction of the nanoparticles dispersed in the base fluid.

Specific Heat Capacity of the Nanofluid:6
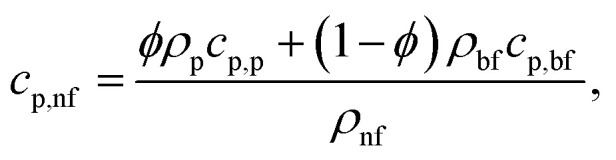
where *c*_p,nf_ denotes the specific heat capacity of the nanofluid. The symbols *c*_p,p_ and *c*_p,bf_ refer to the specific heat capacities of the nanoparticle and the base fluid, respectively.

Viscosity of the Nanofluid:7
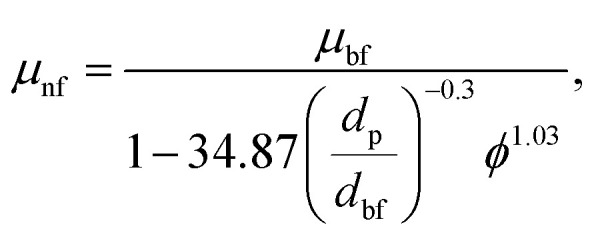
where *µ*_nf_ is the dynamic viscosity of the nanofluid and *µ*_bf_ is the viscosity of the base fluid. *d*_p_ is the diameter of the nanoparticle, while *d*_bf_ is the molecular diameter of the base fluid, calculated as follows:8
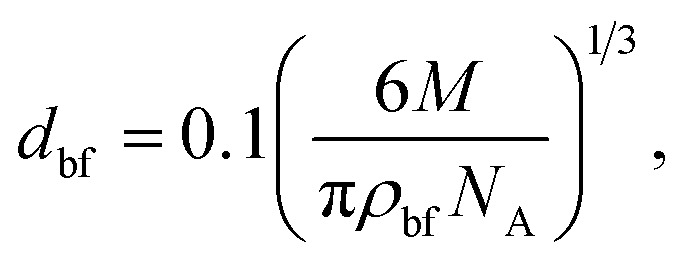
where *M* is the molecular weight of water (0.018015 kg mol^−1^) and *N*_A_ is Avogadro's number (6.022 × 10^23^ mol^−1^).

Thermal Conductivity of the Nanofluid (Corcione Correlation^67^):9
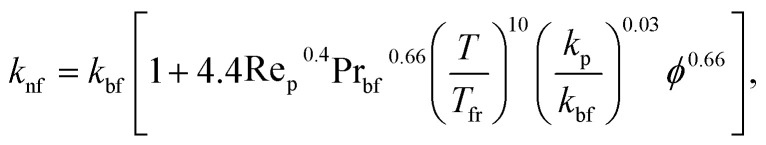
where *k*_nf_ is the thermal conductivity of the nanofluid, and *k*_bf_ and *k*_p_ are the thermal conductivities of the base fluid and the nanoparticle, respectively. *T* is taken as the inlet temperature (300 K), *T*_fr_ is the freezing point of water (273 K), Pr_bf_ is the Prandtl number of the base fluid, and Re_p_ is the particle Reynolds number given as follows:10
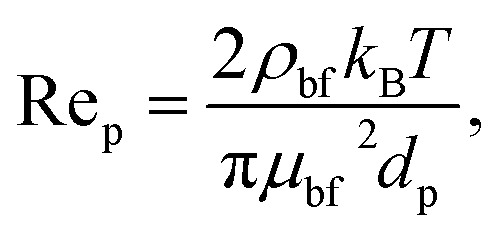
where *k*_B_ is the Boltzmann constant (1.3806 × 10^−23^ J K^−1^). It must be noted that the Corcione model is valid for spherical nanoparticles in the range of 10–150 nm with a concentration ≤5%.

For nanofluids containing graphene (G), graphene oxide (GO), and hexagonal boron nitride (hBN), the nanomaterials are not spherical, but exist in the form of nanoplatelets or 2D flakes. As such, the widely used Corcione model, which assumes spherical particles, may not yield accurate predictions for these types of nanofluids. To address this, the present study utilizes the Hamilton–Crosser model^68^ to estimate the effective thermal conductivity, incorporating the effect of particle shape *via* a sphericity factor. A similar approach has been adopted in previous studies.^69–71^ The remaining thermophysical properties (density, specific heat, and viscosity) were computed using the same equations as used for spherical particles, as these properties are not as strongly affected by shape.

Thermal Conductivity of the Nanofluid (Hamilton–Crosser Model^68^):11
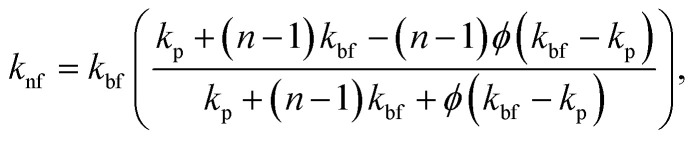
where *n* is the shape factor defined as *n* = 3/*ξ* and *ξ* is the sphericity of the particle. For nanoplatelets, *ξ* is taken as 0.15 in this study, resulting in *n* ≈ 20. This high value of *n* reflects the low sphericity and high aspect ratio of platelet-like particles. A sensitivity analysis was conducted by varying *ξ* between 0.10 and 0.20 around the baseline value of *ξ* = 0.15. In addition, the influence of platelet viscosity modeling was assessed using the Krieger–Dougherty relation ^72^ with intrinsic viscosity and maximum packing fraction values reported in the literature.^73,74^ The detailed results of these sensitivity analyses are presented in Appendix A. The results indicate that variations in cavity wall temperature, thermal performance factor, and thermal effectiveness remain small, confirming that the primary conclusions of this study are not significantly affected by reasonable variations in nanoparticle morphology assumptions.

The Reynolds number and Prandtl number of the nanofluid are given as follows:12
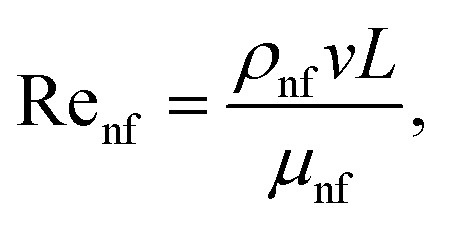
13
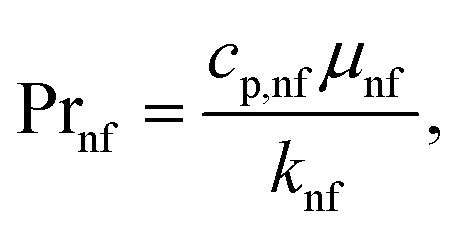
where *v* is the inlet velocity and *L* in the characteristic length (inlet width). The average convective heat transfer coefficient (*h*) is calculated using Newton's law of cooling, which is mathematically represented as follows:14
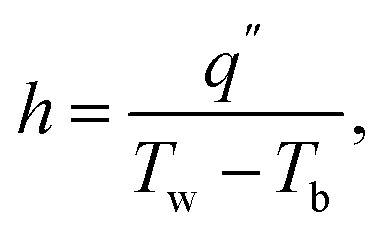
where *q*″ represents the heat flux, *T*_w_ denotes the mean temperature of the heated wall, and *T*_b_ indicates the average working fluid bulk temperature. To evaluate the average Nusselt number (Nu), the following relation is used:15
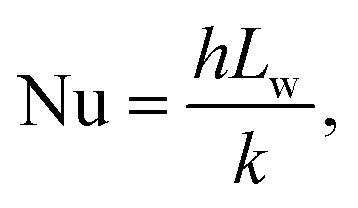
where *L*_w_ is the length of the cavity wall. Table 2 presents the thermophysical properties of water and the nanoparticles utilized in this study. The thermal effectiveness and thermal performance factor (TPF) are calculated as follows:16
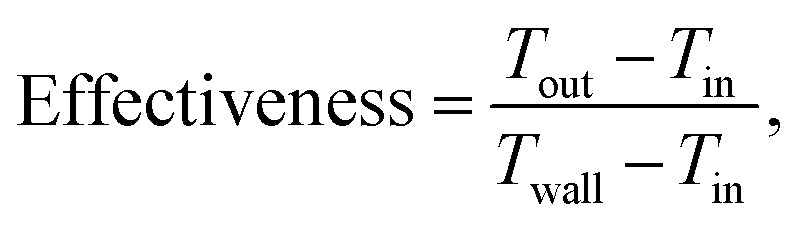
17
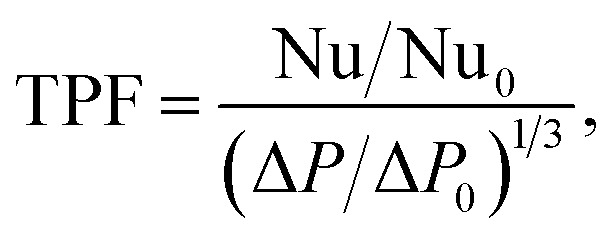
where *T*_out_, *T*_wall_, and *T*_in_ are the outlet temperature, cavity wall temperature, and inlet temperature, respectively. Nu and Δ*P* refer to the Nusselt number and pressure drop obtained using the nanofluid, while Nu_0_ and Δ*P*_0_ denote the Nusselt number and pressure drop calculated for the base fluid (water) under identical operating conditions. The thermal performance factor defined in eqn (17) is a widely used thermohydraulic evaluation index that combines heat-transfer enhancement with the associated pressure-drop penalty.^75–78^

**Table 2:** Thermophysical properties of water and the nanoparticles used in this study

Material	Density *ρ* (kg m^−3^)	Specific heat *c*_p_ (J kg^−1^ K^−1^)	Thermal conductivity *k* (W m^−1^ K^−1^)
Water (base fluid)	998.8	4182	0.611
Aluminum (Al)	2700	900	237
Aluminum oxide (Al_2_O_3_)	3970	765	40
Copper (Cu)	8933	385	400
Copper oxide (CuO)	6000	540	18
Graphene (G)	2250	2100	2500
Graphene oxide (GO)	1800	670	5
Hexagonal boron nitride (hBN)	2200	700	60

### 2.3. Numerical method

**Fig. 1:**
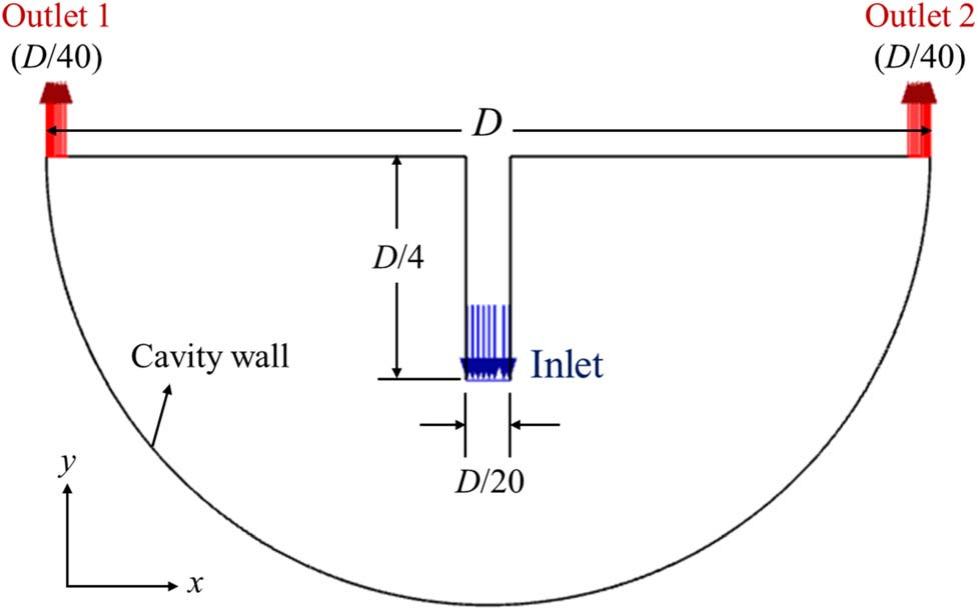
Computational domain illustrating the curved cavity geometry along with the locations of the inlet and outlets.

The computational domain consists of a two-dimensional, semi-circular cavity with a top-centered vertical inlet and two symmetric outlets located at both ends of the horizontal top wall, as illustrated in Fig. 1. The diameter of the semi-circular cavity is denoted by *D*, while the inlet width is set to *D*/20 and extends vertically by *D*/4. This geometric configuration is representative of flow conditions found in electronics cooling applications and curved thermal ducts. A uniform velocity inlet boundary condition was applied at the vertical inlet at a constant temperature of 300 K, corresponding to a Reynolds number of 2500 based on the inlet width and water as the base fluid. This Reynolds number indicates a transitional flow regime, making the choice of the transition SST *K–ω* model particularly suitable for capturing near-wall effects and transition to turbulence within the curved cavity. At the cavity wall, a constant heat flux boundary condition of *q*″ = 50 000 W m^−2^ was imposed to simulate an externally heated surface, and all other walls were considered adiabatic. The two outlets were modeled as pressure outlets with zero-gauge pressure, allowing for symmetric discharge from the domain. The working fluid was either pure water or one of the seven different water-based nanofluids, each consisting of dispersed nanoparticles (Al, Al_2_O_3_, Cu, CuO, G, GO, and hBN) with a fixed particle size of 10 nm. Nanoparticle volume concentrations of 0%, 0.5%, 1%, 3%, and 5% were considered.

Although nanofluids are inherently two-phase systems, previous studies^79,80^ have shown that they exhibit a behavior more similar to that of pure fluids than that of liquid–solid mixtures. Therefore, in this study, a single-phase model was adopted, treating the nanofluid as a continuum with effective thermophysical properties derived from those of the base fluid and nanoparticles. All nanofluids were also assumed to behave as classical Newtonian fluids, consistent with the single-phase approach.^81,82^ The flow was solved under steady-state conditions using the pressure-based solver in ANSYS Fluent. The SIMPLE algorithm was applied for pressure–velocity coupling. All spatial discretization schemes were set to second-order accuracy, and gradients were computed using the least squares cell-based method. A convergence criterion of 10^−6^ was applied to all residuals. Mesh refinement was focused near the cavity wall to resolve steep gradients in the boundary layer.

The use of a two-dimensional, steady-state formulation is justified by the geometric symmetry of the semi-circular cavity and the study's primary objective, *i.e.*, to compare the steady thermohydraulic performance of various nanofluids under identical boundary conditions. Although minor secondary motions may exist in curved geometries, prior studies^79,80,82^ have shown that for moderate Reynolds numbers and symmetric configurations, two-dimensional steady simulations capture the mean flow and heat transfer characteristics with good agreement to full three-dimensional or transient results. Moreover, the SST *K-ω* turbulence model effectively accounts for near-wall and transitional effects within a steady framework. Since both the inlet velocity and wall heat flux are constant, the system evolves toward a quasi-steady-state thermal equilibrium, making the steady-state assumption physically representative of the time-averaged behavior. This modeling approach ensures an appropriate balance between physical fidelity and computational efficiency for the multi-nanofluid comparative analysis performed in this study.

The key assumptions adopted in developing the mathematical model are summarized as follows.

• The flow is considered two-dimensional and incompressible.

• The nanofluid is treated as a single-phase, Newtonian fluid with uniform and constant thermophysical properties.

• The nanoparticles are uniformly dispersed, maintaining consistent size and shape throughout the domain.

• Thermal equilibrium is assumed between the nanoparticles and the base fluid, forming a homogeneous mixture.

• The effective thermophysical properties of the nanofluid are considered isotropic and temperature-independent. This assumption is justified by the small bulk-fluid temperature rise observed in the present simulations, with a maximum variation of approximately 0.86 K, which results in negligible changes in fluid properties.

• The top surface of the cavity is assumed to be adiabatic.

These assumptions are consistent with those adopted in several prior studies on nanofluids.^83–86^ Since the primary objective of the present work is a comparative assessment of different nanofluids under identical operating conditions, the use of these assumptions is considered appropriate and does not affect the relative performance trends reported.

### 2.4 Mesh independence study

**Fig. 2:**
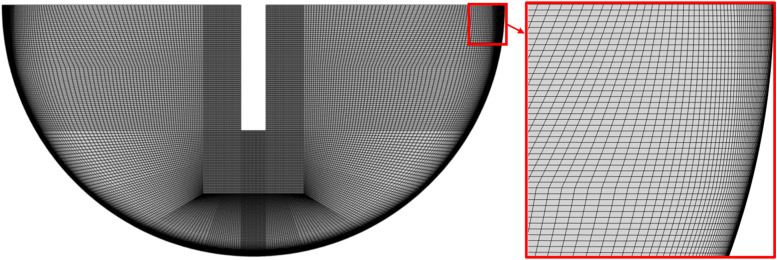
Structured computational mesh with a refined spacing near the heated cavity wall.

To establish confidence in the accuracy of the numerical simulations, a mesh independence study was performed using four different mesh densities, as summarized in Table 3. All meshes were generated as structured quadrilateral grids, with a refined mesh spacing near the cavity wall to adequately resolve the steep velocity and thermal gradients expected in the boundary layer regions due to the applied heat flux. A visual representation of the baseline mesh used for the simulations is shown in Fig. 2. The refinement near the heated cavity wall was specifically designed to ensure that the dimensionless wall distance (*y*^+^) remained below 1 across the entire heated surface, thereby fully resolving the viscous sublayer. This resolution satisfies the requirements of the SST *K–ω* model, which employs a low-Reynolds-number formulation near the wall without relying on wall functions, ensuring the accurate prediction of near-wall flow and thermal gradients.

**Table 3:** Mesh independence results showing variation in the heat transfer coefficient and cavity temperature for different mesh densities

Mesh designation	No. of elements	Heat transfer coefficient (W m^−2^ K^−1^)	Difference in heat transfer coefficient (%)	Cavity temperature (K)	Difference in cavity temperature (%)
Coarse 1	8960	3269.5	8.50	316.70	0.41
Coarse 2	21 968	3139.8	4.20	317.25	0.23
Baseline	34 304	3035.6	0.74	317.82	0.05
Fine	84 048	3013.3	—	317.99	—

The comparison was based on two key output parameters: the average convective heat transfer coefficient at the cavity wall and the mean cavity wall temperature. As presented in Table 3, the transition from the Coarse 1 mesh to the Fine mesh results in a progressive reduction in the deviation of both the heat transfer coefficient and cavity temperature from Fine mesh values. Notably, the differences become marginal beyond the Baseline mesh, indicating mesh-independent behavior. Specifically, the difference in heat transfer coefficient between the Baseline and Fine meshes was only 0.74%, while the difference in average cavity wall temperature was a negligible 0.05%. Given this minimal variation and the significantly reduced computational cost compared to the Fine mesh, the Baseline mesh was selected for all subsequent simulations. This mesh provides a practical balance between the solution accuracy and the computational efficiency.

### 2.5 Model validation

**Fig. 3:**
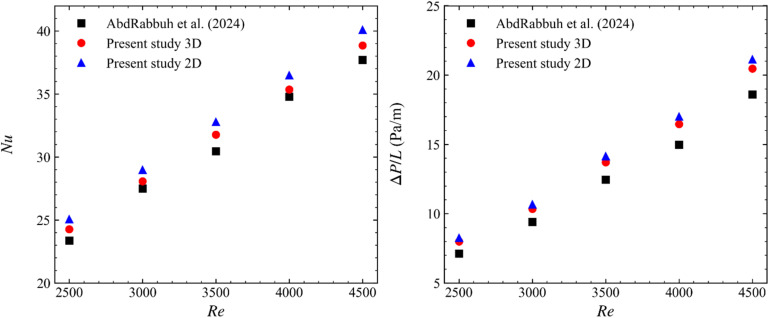
Validation of the present numerical model against the results of AbdRabbuh *et al.*^38^ for a circular annular pipe with water as the working fluid at various Reynolds numbers: (left) average Nusselt number (Nu) and (right) pressure drop per unit pipe length (Δ*P/L*).

To ensure the accuracy and reliability of the numerical model developed in this study, model validation was performed by replicating the experimental results of AbdRabbuh *et al.*,^38^ who investigated the thermal and hydrodynamic performance of water and graphene nanofluids in a fully circular pipe under transitional flow conditions. The experimental configuration of AbdRabbuh *et al.*^38^ was replicated numerically using distilled water as the working fluid. The same thermophysical properties, pipe geometry, and inlet conditions were adopted, covering Reynolds numbers in the range of 2500–4500. Two CFD models were implemented: (i) a full 3D pipe model and (ii) a 2D axisymmetric representation of the same geometry. In both cases, identical boundary conditions and the transition *SST K*–*ω* turbulence model were employed. The numerical results were compared with the experimental data in terms of the average Nusselt number (Nu) and the pressure drop per unit pipe length (Δ*P*/*L*). As shown in Fig. 3, both the 3D and 2D axisymmetric simulations reproduce the experimental trends very well over the entire Reynolds number range. The 3D model exhibits a very close agreement with the measurements, whereas the 2D axisymmetric model slightly overpredicts both Nu and Δ*P/L*. The observed deviations can be attributed to several factors. First, while the reference study by AbdRabbuh *et al.*^38^ was experimental in nature, the present work employs a CFD simulation, which inherently involves modeling assumptions such as idealized boundary conditions, mesh discretization, and turbulence model approximations. Additionally, experimental uncertainties such as slight variations in material properties or pipe surface roughness could contribute to discrepancies. The numerical model, on the other hand, assumes smooth walls and fully developed flow conditions. Despite these differences, the overall agreement between the datasets confirms the reliability of the current numerical framework for predicting thermal and hydrodynamic behavior in similar flow configurations.

**Fig. 4:**
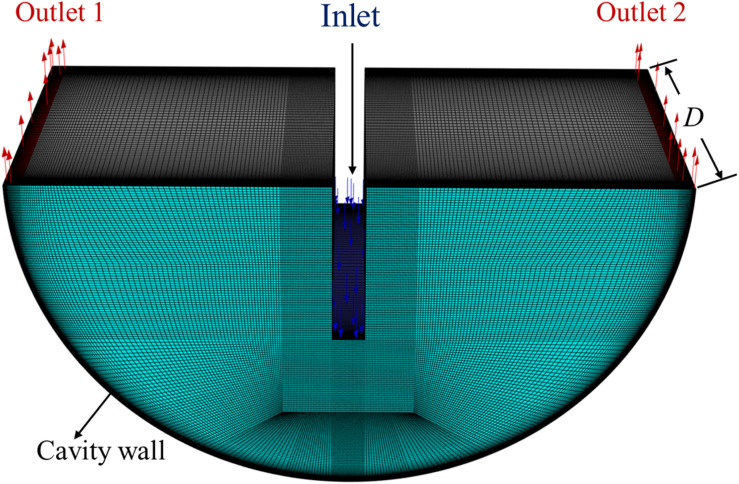
3D semi-circular cavity geometry and computational mesh used to assess the validity of the 2D numerical model.

In addition to the comparison with the external experimental data, the present 2D model was tested against a full 3D simulation of the semi-circular cavity configuration. The 3D domain, shown in Fig. 4, was generated by extruding the 2D geometry in the spanwise direction while maintaining the same boundary conditions. The average heat transfer coefficient at the heated wall, cavity-average temperature, outlet bulk temperature, and total pressure drop were evaluated from both simulations. As summarized in Table 4, the maximum deviation between the 2D and 3D predictions is 2.84% for the heat transfer coefficient, whereas the differences in cavity temperature, outlet temperature, and pressure drop remain below 1%. These discrepancies are well within the typical numerical uncertainty bounds for CFD simulations, and confirm that the 2D formulation captures the dominant thermohydraulic behavior of the semi-circular cavity. Therefore, the 2D model is adopted for the subsequent parametric investigation of water-based nanofluids, providing an appropriate balance between the computational efficiency and the predictive accuracy.

**Table 4:** Comparison of the key thermohydraulic parameters predicted by the 2D and 3D models for water at Re = 2500 in the semi-circular cavity

Parameter	2D model	3D model	Difference (%)
Heat transfer coefficient (W m^−2^ K^−1^)	3035.64	3121.88	2.84
Cavity temperature (K)	317.82	316.66	0.365
Outlet temperature (K)	300.82	300.80	0.00665
Pressure drop (Pa)	98.43	99.10	0.681

## 3. Results and discussion

This section presents and discusses the effect of the inlet position and the thermohydraulic performance of various nanofluids in a semi-circular cavity. The analysis encompasses seven water-based nanofluids: Al, Al_2_O_3_, Cu, CuO, graphene (G), graphene oxide (GO), and hexagonal boron nitride (hBN), with volume concentrations ranging from 0.5% to 5%.

### 3.1. Effect of inlet position

To assess the influence of geometric modification on the flow behavior and heat transfer efficiency, a preliminary investigation was conducted to evaluate the effect of inlet position within the semi-circular cavity. Three configurations were tested using water as the working fluid: (i) a top inlet, (ii) an inlet placed at a quarter depth from the top of the cavity, and (iii) an inlet positioned at half depth from the top of the cavity. All other parameters, including the inlet velocity (corresponding to Re = 2500), inlet temperature (300 K), and cavity heating conditions, were kept constant.

**Fig. 5:**
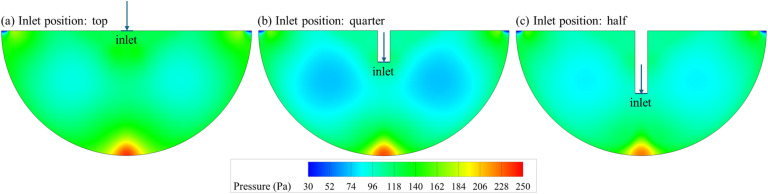
Pressure contour maps for different inlet positions of (a) top, (b) quarter-depth, and (c) half-depth.

**Fig. 6:**
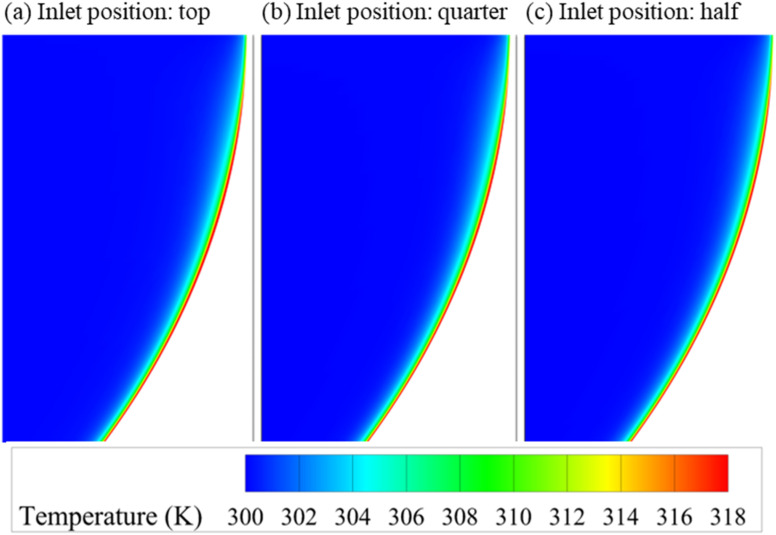
Temperature contour maps inside the cavity for different inlet positions of (a) top, (b) quarter-depth, and (c) half-depth.

Table 5 summarizes the results in terms of average convective heat transfer coefficient, mean cavity temperature, outlet temperature, and pressure drop. While the changes in thermal parameters are relatively minor, a clear trend emerges with the increasing inlet depth: the average heat transfer coefficient decreases from 3274.2 W m^−2^ K^−1^ (top) to 3035.6 W m^−2^ K^−1^ (half-depth), accompanied by a rise in mean cavity temperature and a slight increase in outlet temperature. Conversely, the pressure drop decreases significantly, from 126.9 Pa (top inlet) to 98.4 Pa (half-depth inlet). This trade-off reflects a shift in flow dynamics within the cavity. As the inlet is placed deeper into the cavity, the incoming jet is less effective at sweeping along the heated cavity wall, resulting in diminished convective transport and reduced thermal performance. However, this configuration minimizes the pressure drop due to the shorter acceleration path and more favorable flow development. These observations are supported by the pressure contours in Fig. 5 and temperature fields in Fig. 6. The top-inlet configuration shows a more concentrated high-pressure region in front of the inlet. In contrast, the half-depth inlet promotes a gentler flow, reducing flow resistance but also limiting thermal extraction.

**Table 5:** Effect of inlet position on the average convective heat transfer coefficient, cavity temperature, outlet temperature, and pressure drop at Re = 2500 using water

Inlet position	Heat transfer coefficient (W m^−2^ K^−1^)	Cavity temperature (K)	Outlet temperature (K)	Pressure drop (Pa)
Top	3274.2	316.58	300.77	126.92
Quarter	3262.06	316.72	300.78	110.59
Half	3035.64	317.82	300.82	98.43

Given the significant disparity in pressure drop (over 22% reduction from top to half-depth) and the modest loss in thermal performance (∼7% reduction in convective heat transfer coefficient and only 0.4% drop in cavity temperature), the half-depth inlet was selected for subsequent nanofluid simulations. This geometry provides a more energy-efficient platform, particularly important considering the added viscosity and associated pressure penalties introduced by nanoparticle-laden fluids.

### 3.2 Thermal performance

**Fig. 7:**
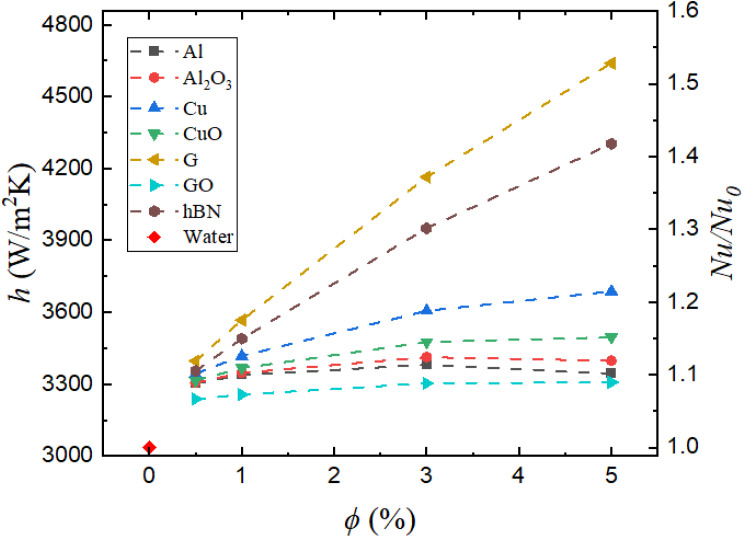
Variation in the convective heat transfer coefficient (*h*) and normalized Nusselt number (Nu/Nu_0_) with the nanoparticle volume concentration (*ϕ*).

Fig. 7 presents the variation in the convective heat transfer coefficient (*h*) and the normalized Nusselt number (Nu/Nu_0_), where Nu_0_ corresponds to the Nusselt number obtained using the base fluid (water). A clear enhancement in thermal performance is observed upon the introduction of nanoparticles, especially with the increasing volume concentration (*ϕ*). Similar enhancement trends with the increasing nanoparticle volume fraction have been widely reported in experimental and numerical studies of nanofluid convection flows.^19,26,37,87,88^

Among all nanofluids, graphene/water shows the most significant improvement, with the convective heat transfer coefficient reaching ∼4700 W m^−2^ K^−1^ at 5% concentration, corresponding to an increase of 53% over water. The normalized Nusselt number also peaks for graphene at 1.53, highlighting its excellent capacity to augment convective heat transport. The second-best performer is hBN/water, which achieves a convective heat transfer coefficient of 4303 W m^−2^ K^−1^, representing a 42% enhancement over water. These improvements are attributed to their high thermal conductivities (as shown in Table 6) and unique platelet morphology, which promotes rapid in-plane conduction and increases the surface area for heat exchange.

**Table 6:** Thermophysical properties of the water-based nanofluids at 5% volume concentration

Nanofluid	Density *ρ*_nf_ (kg m^−3^)	Specific heat *c*_p,nf_ (J kg^−1^ K^−1^)	Thermal conductivity *k*_nf_ (W m^−1^ K^−1^)
Water (base fluid)	998.8	4182	0.611
Al/water	1083.8	3773.2	0.764
Al_2_O_3_/water	1147.3	3590.8	0.756
Cu/water	1395.5	2966.7	0.7664
CuO/water	1248.8	3307.1	0.7526
G/water	1061.3	3961.3	1.2509
GO/water	1038.8	3877.7	0.7746
hBN/water	1058.8	3820.2	1.1397

Interestingly, although copper (Cu) nanoparticles possess a significantly higher intrinsic thermal conductivity (∼400 W m^−1^ K^−1^) than hexagonal boron nitride (hBN) nanoparticles (∼60 W m^−1^ K^−1^), the Cu/water nanofluid exhibits inferior thermal performance. At 5% concentration, the convective heat transfer coefficient for Cu/water is 3687 W m^−2^ K^−1^, which is notably lower than that of hBN/water. This is because the effective thermal conductivity of the Cu/water nanofluid at 5% concentration (0.7664 W m^−1^ K^−1^) is actually lower than that of hBN/water (1.1397 W m^−1^ K^−1^), highlighting the importance of nanoparticle dispersion and interaction with the base fluid over intrinsic properties alone. CuO/water, though less conductive, exhibits similar trends and begins to plateau after 3%, suggesting diminishing returns due to increased flow resistance and energy dissipation.

Al/water and Al_2_O_3_/water demonstrate relatively low enhancements. Al/water, while possessing good intrinsic conductivity, is chemically unstable and prone to agglomeration in aqueous media, which may reduce its effective surface area. Al_2_O_3_/water, though more stable, exhibits a modest gain (3413 W m^−2^ K^−1^ at 3%) that flattens beyond 3%. This plateau behavior indicates that viscous effects begin to offset thermal gains at higher concentrations. The poorest performer is GO/water, which shows minimal improvement. Although GO/water is chemically more stable than G/water, its significantly lower conductivity outweighs any stability benefit, leading to a convective heat transfer coefficient value of 3308 W m^−2^ K^−1^ at 5%.

The enhancement trends are non-linear, suggesting that beyond certain concentration thresholds, further nanoparticle addition either provides minimal benefit or may even deteriorate thermal performance due to increased viscosity, reduced effective Reynolds number, and potential onset of micro-scale flow instabilities.

**Fig. 8:**
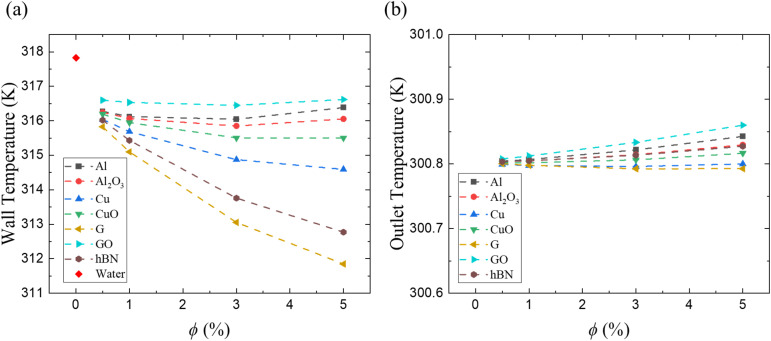
(a) Average cavity wall temperature and (b) average outlet nanofluid temperature as a function of the nanoparticle volume concentration (*ϕ*).

Fig. 8(a) displays the average cavity wall temperature for all nanofluids, serving as an indirect indicator of heat transfer efficiency. Lower wall temperatures indicate better convective removal of heat from the wall–fluid interface. At 5% volume concentration, Graphene/water and hBN/water significantly reduce the wall temperature to 311.8 K and 312.7 K, respectively, compared to water's 317.8 K. These reductions are consistent with the convective heat transfer coefficient and Nu/Nu_0_ trends in Fig. 7. For other nanofluids such as Cu/water, CuO/water, and Al_2_O_3_/water, the temperature drops are more modest (314.6 K, 315.5 K, and 316.1 K, respectively), while GO/water yields negligible reduction (316.6 K). Notably, wall temperature decreases significantly up to 3% concentration for most nanofluids, after which a plateau or slight increase is observed. This is due to increasing viscosity which decreases the effective Reynolds number. The interplay of reduced temperature and increasing pumping demands at high concentrations is a central trade-off in nanofluid optimization.

Fig. 8(b) presents the corresponding average outlet nanofluid temperature as a function of nanoparticle volume concentration. The outlet temperature increases slightly with the increase in concentration for most nanofluids due to improved heat absorption from the heated cavity wall. However, the overall temperature rise remains small because of the relatively high flow rate associated with the transitional Reynolds number (Re = 2500). The maximum outlet bulk temperature increase observed is approximately 0.86 K relative to the inlet temperature (300 K) for the GO/water nanofluid at 5% concentration. This limited temperature rise confirms that the working fluid experiences only a modest thermal gain while effectively transporting heat away from the cavity wall.

**Fig. 9:**
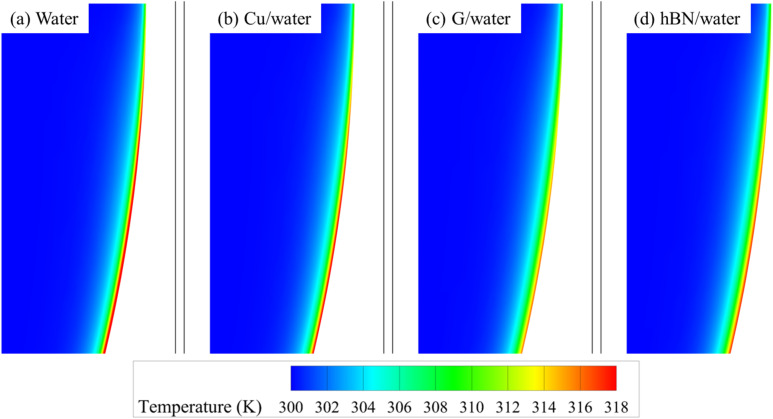
Temperature contour maps within the semi-circular cavity for (a) water, (b) Cu/water, (c) G/water, and (d) hBN/water nanofluids at 5% concentration.

The temperature contours presented in Fig. 9 provide visual support for the quantitative analysis. Nanofluids such as Cu/water, G/water, and hBN/water demonstrate noticeably thicker thermal boundary layers and more uniform thermal diffusion along the heated cavity wall than the base fluid (water). Notably, the G/water and hBN/water nanofluids exhibit steeper temperature gradients near the wall, signifying enhanced local heat flux and more efficient thermal boundary layer transport. Moreover, the high-temperature zone typically observed adjacent to the cavity wall in the water and Cu/water cases is significantly suppressed in the G/water configuration. This reduction in thermal hotspots reflects the superior thermal conductivity and convective transport of graphene-enhanced nanofluids, leading to more effective and evenly distributed heat extraction.

**Fig. 10:**
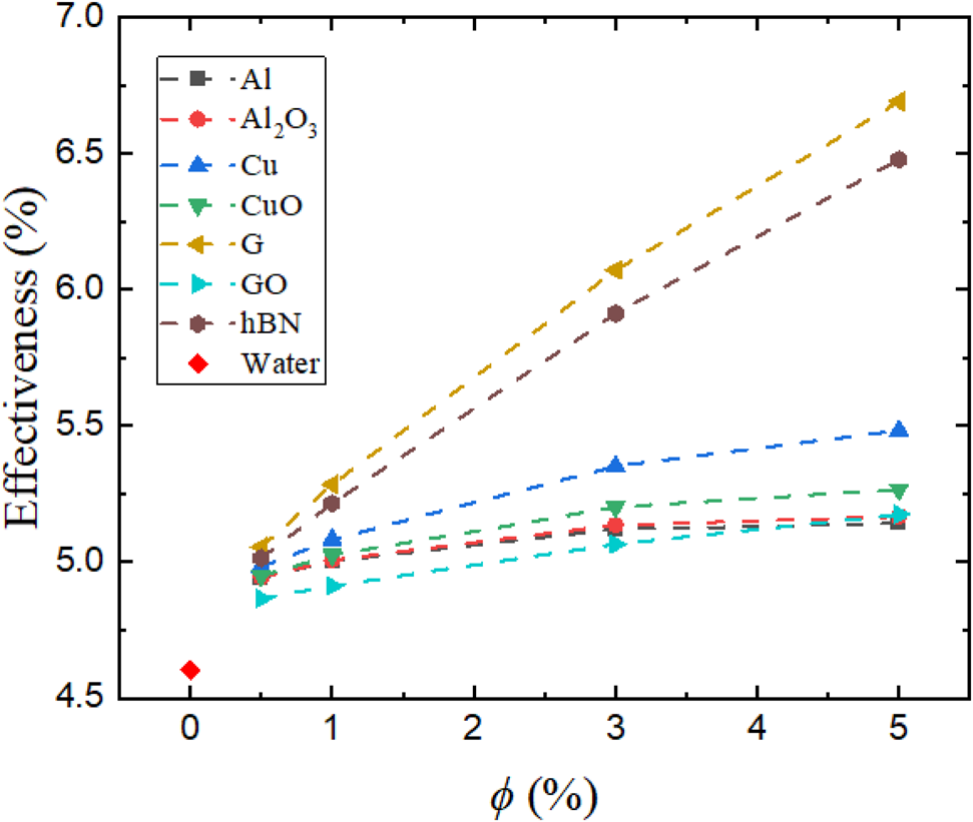
Thermal effectiveness of the nanofluids as a function of the nanoparticle volume concentration (*ϕ*).

Fig. 10 presents the thermal effectiveness, computed using eqn (16), which quantifies the extent to which the cooling fluid utilizes the available thermal potential for heat absorption. Water shows a baseline effectiveness of 4.6%. G/water and hBN/water nanofluids achieve the highest values (6.7% and 6.5%, respectively) at 5% volume concentration. Cu/water and CuO/water provide intermediate effectiveness (5.3% and 5.5%, respectively), while Al/water, Al_2_O_3_/water, and GO/water remain below 5.2%.

This trend directly reflects the cumulative effect of enhanced convective heat transfer coefficient, reduced wall temperature, and favorable nanoparticle properties. Thermal effectiveness shows saturation behavior for Al/water and Al_2_O_3_/water beyond 3%, mirroring earlier thermal indicators. Al/water, Al_2_O_3_/water, and GO/water show low performance due to their limited heat transfer ability.

### 3.3 Pressure drop and pumping power

**Fig. 11:**
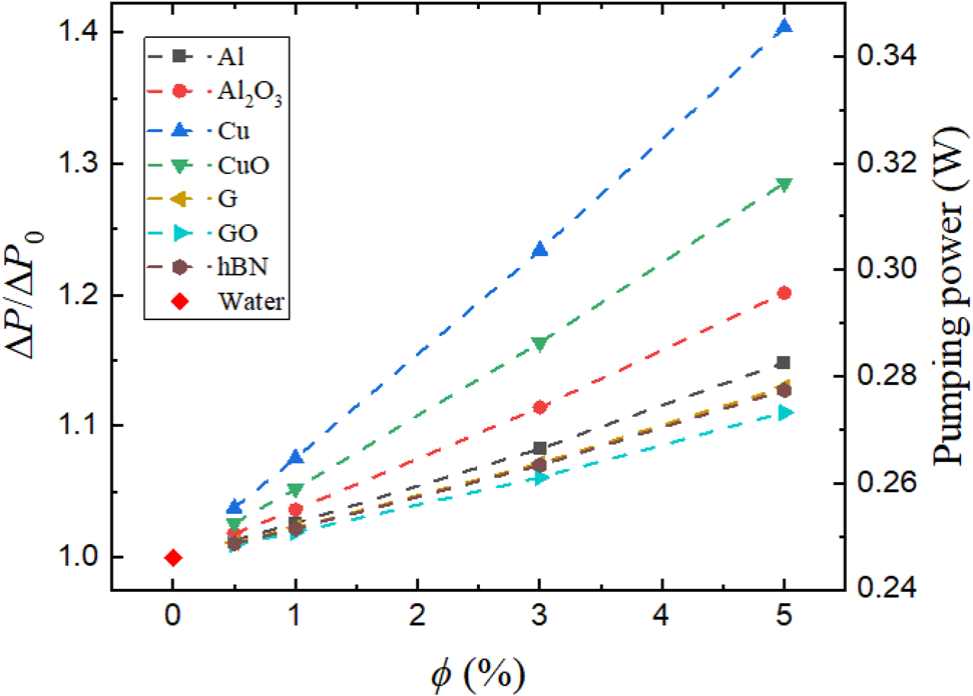
Normalized pressure drop (Δ*P*/Δ*P*_*0*_) and pumping power variations with the nanoparticle volume concentration (*ϕ*).

The impact of nanoparticle addition on flow resistance is comprehensively evaluated in Fig. 11, which presents both the normalized pressure drop (Δ*P*/Δ*P*_0_) and the corresponding pumping power required for each nanofluid.

Among all the tested nanofluids, the Cu/water nanofluid exhibits the highest pressure drop, reaching 1.40 times that of the base fluid at a 5% volume concentration. This is closely followed by CuO/water, which shows a normalized pressure drop 1.29 times that of the base fluid. These elevated values are primarily attributed to the high densities of copper-based nanofluids, which increase the inertial resistance to flow. As a result, wall shear stresses are significantly elevated, necessitating greater pumping power to maintain a constant flow rate. In contrast, G/water and hBN/water nanofluids exhibit only moderate increases in pressure drop, with values around 1.13 times that of water. This indicates a more favorable thermohydraulic balance, where substantial thermal benefits are achieved without imposing excessive hydraulic penalties. This behavior is due to the unique platelet-like morphology and high aspect ratio of these nanoparticles. Their lower densities compared to metallic nanoparticles also help maintain the fluid momentum and reduce the overall flow resistance.

The associated pumping power requirements, shown on the secondary *y*-axis of Fig. 11, follow a similar trend. Cu/water and CuO/water nanofluids require the highest input energy, reaching 0.345 W and 0.316 W, respectively, at 5% concentration. G/water and hBN/water, despite their high thermal performance, require less than 0.28 W, confirming their suitability for applications where both thermal enhancement and energy efficiency are desired. Al/water and GO/water nanofluids not only exhibit low pumping power demands but also provide limited thermal enhancement, as discussed in Fig. 7 and 8, thereby reducing their overall effectiveness in performance-critical systems. This shows the importance of balancing thermal gains with hydrodynamic costs. Nanofluids such as G/water and hBN/water offer the most promising profiles, achieving meaningful thermal improvements while maintaining manageable increases in pressure drop and pumping power. Conversely, while copper-based nanofluids may enhance heat transfer, their high hydraulic demands could limit their practicality in energy-sensitive or compact flow systems.

**Fig. 12:**
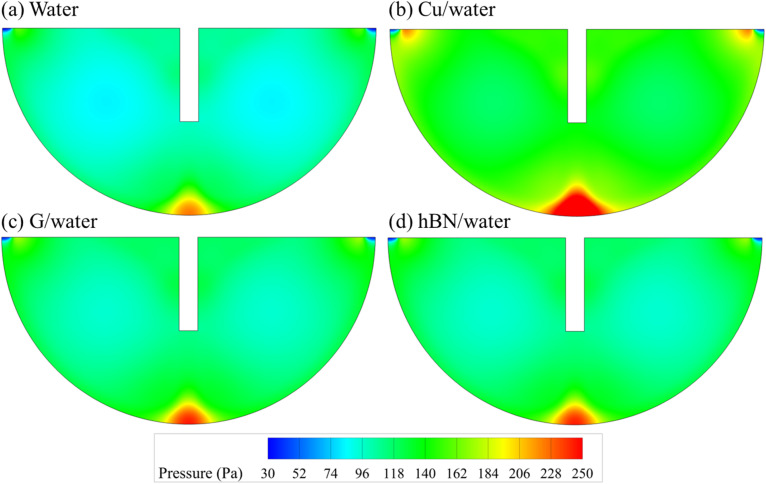
Pressure contour maps inside the cavity for (a) water, (b) Cu/water, (c) G/water, and (d) hBN/water nanofluids at 5% concentration.

The pressure contours in Fig. 12 support the quantitative findings. For water, GO/water, and hBN/water, the high-pressure zones are localized near the bottom wall directly in front of the inlet, while the pressure within the cavity remains relatively low. In contrast, Cu/water displays a more pronounced and expanded high-pressure region, accompanied by elevated pressure levels throughout the cavity. This distribution reflects greater flow resistance and is consistent with the higher pressure drop and pumping power observed for Cu-based nanofluids.

### 3.4. Thermal performance factor

**Fig. 13:**
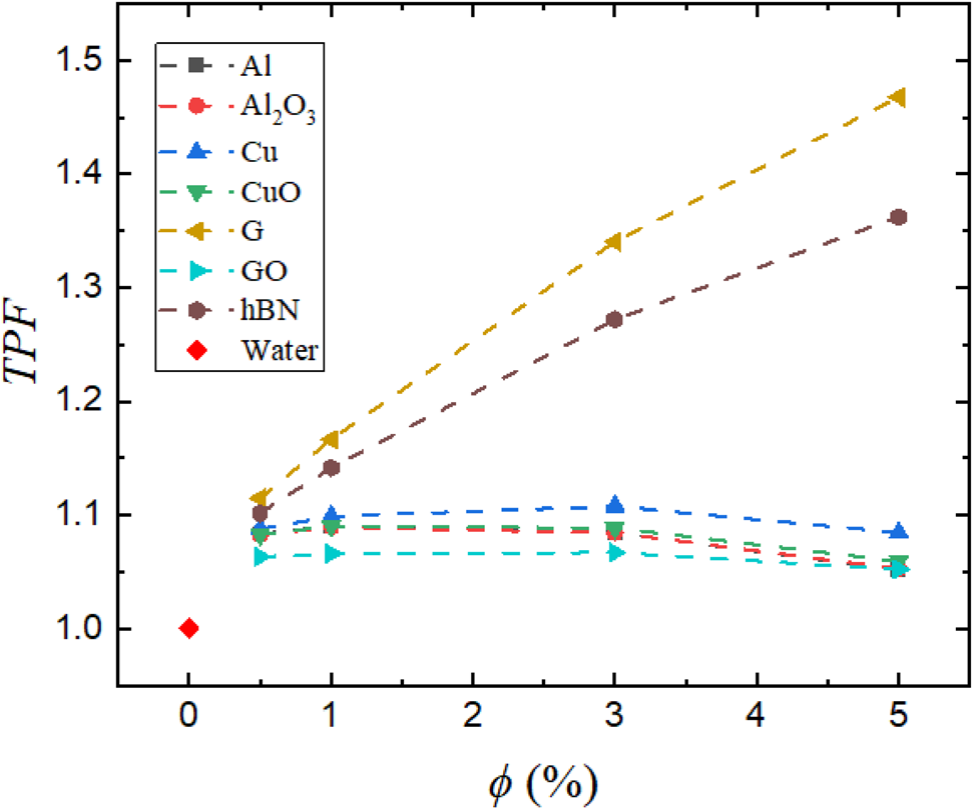
Plot of the thermal performance factor (TPF) of nanofluids *versus* nanoparticle volume concentration (*ϕ*).

Fig. 13 shows the Thermal Performance Factor (TPF) for the nanofluids used in this study. It reflects the net benefit of nanofluid use by combining heat transfer gain and flow penalty into a single metric, and is calculated according to eqn (17).

Among all the tested nanofluids, graphene (G/water) demonstrates the most favorable performance, achieving a TPF of approximately 1.47 at 5% concentration. This highlights graphene's ability to deliver substantial thermal enhancement while incurring only a modest increase in pressure drop. hBN/water follows closely with a TPF of ∼1.36, reinforcing its potential as a high-efficiency coolant. The high aspect ratio and anisotropic thermal conductivity of these nanoplatelet structures contribute to significant heat transfer improvements without causing substantial increases in flow resistance.

In contrast, Cu/water, while thermally effective as seen in earlier metrics, records a lower TPF of ∼1.08 at 5%. This drop in performance is directly linked to its higher pressure drop, which consequently reduces the overall benefit when energy costs are considered. CuO/water, Al/water, Al_2_O_3_/water, and GO/water nanofluids exhibit TPF values near 1.05, suggesting that the thermal gains offered by these nanofluids are nearly offset by the accompanying increase in pumping power. The combined results from Fig. 4–12 highlight that selecting a nanofluid is a multi-objective optimization task.

## 4. Limitations and future work

The present work is based on a two-dimensional, steady-state, single-phase approach with temperature-independent thermophysical properties. Although the present analysis was conducted at Re = 2500, previous experimental and numerical studies in the transitional regime (Re ≈ 2000–4000) have shown that increasing the Reynolds number generally enhances convective heat transfer for both base fluids and nanofluids while preserving similar relative performance trends among different nanofluids.^89,90^ Formulation-specific effects such as surfactants or nanoparticle surface functionalization, which may influence dispersion stability and viscosity in practical nanofluids, were not explicitly considered in the present numerical framework. While these assumptions enable a clear comparative evaluation among different nanofluids with manageable computational cost, they may not capture transient, three-dimensional, or particle–fluid interaction effects that can occur in real systems. Future studies should therefore extend the analysis to three-dimensional and transient domains, incorporating two-phase or particle-tracking models and temperature-dependent properties to more accurately represent nanofluid behavior under dynamic thermal loads. Experimental validation under similar geometric and operating conditions is also recommended to further substantiate the numerical predictions and refine the modeling framework for practical applications.

## 5. Conclusions

In this study, a two-dimensional numerical investigation was conducted to evaluate the thermohydraulic performance of various water-based nanofluids within a semi-circular cavity under transitional flow conditions. Using the transition SST *K–ω* model in ANSYS Fluent, simulations were performed at a Reynolds number of 2500. The base fluid was water, and nanoparticles included Al, Al_2_O_3_, Cu, CuO, G, GO, and hBN, introduced at volume concentrations of 0.5%, 1%, 3%, and 5%. Thermophysical properties were modeled using established empirical correlations appropriate for spherical and platelet-shaped particles. The key findings of this study are as follows.

• Graphene/water and hBN/water nanofluids exhibited the highest thermal enhancement, achieving heat transfer coefficients up to 53% and 42% higher than water, respectively. Their superior performance is attributed to high thermal conductivity, anisotropic morphology, and moderate flow resistance.

• Copper-based nanofluids (Cu/water and CuO/water) demonstrated reasonable thermal improvement but were hindered by significant increases in pressure drop and pumping power, limiting their applicability in energy-sensitive systems. Al/water and Al_2_O_3_/water nanofluids, despite offering moderate conductivity, showed only marginal improvements in heat transfer.

• Graphene oxide (GO), while stable in water, offered limited thermal benefits, and its performance plateaued at higher concentrations, underscoring the importance of balancing stability with conductive potential.

• The thermal performance factor identified G/water and hBN/water as the most efficient nanofluids, with TPF values of 1.47 and 1.36, respectively, at 5% concentration, demonstrating an optimal trade-off between thermal gain and hydraulic penalty.

• Temperature and pressure contours supported the quantitative results, showing improved thermal uniformity and reduced hotspot formation for high-performance nanofluids like G/water and hBN/water, while highlighting elevated flow resistance in Cu-based nanofluids.

These results demonstrate that nanofluid selection is a multi-objective optimization problem, requiring a balance between thermal conductivity, viscosity, stability, and nanoparticle morphology. The semi-circular cavity results presented here are particularly relevant for compact thermal systems, such as microchannel coolers, electronic device heat sinks, and solar thermal collectors, where both heat transfer and flow efficiency are paramount.

## Conflicts of interest

There are no conflicts to declare.

## A. Appendices

### A1. Sensitivity analysis of platelet morphology and viscosity model

#### A1.1. Sensitivity to nanoparticle sphericity in the Hamilton–Crosser model

The Hamilton–Crosser model accounts for particle shape through the sphericity parameter (*ξ*). Since graphene (G), graphene oxide (GO), and hBN nanoparticles exhibit platelet-like morphologies, the assumed sphericity may influence the predicted effective thermal conductivity. In this study, a baseline value of *ξ* = 0.15 was adopted, and a sensitivity analysis was performed by varying *ξ* between 0.10 and 0.20. The resulting variations in cavity wall temperature, thermal performance factor (TPF), and thermal effectiveness at *ϕ* = 5% are summarized in Table 7. The results indicate that the predicted thermohydraulic performance is not significantly sensitive to the assumed sphericity. For G/water and hBN/water nanofluids, the maximum change in cavity wall temperature is below 0.51%, while thermal effectiveness and TPF vary by less than approximately 14% and 16%, respectively. For the GO/water nanofluid, the sensitivity is even smaller, with TPF changes below 1.3% and wall temperature variations below 0.07%. These results indicate that the overall conclusions regarding the superior performance of platelet-based nanofluids do not significantly change within a realistic range of platelet morphologies.

**Table 7:** :Sensitivity of the thermohydraulic performance of platelet-based nanofluids to the particle sphericity (*ξ*) in the Hamilton–Crosser thermal conductivity model at 5% volume fraction

Nanofluid (*ϕ* = 5%)	Sphericity (*ξ*)	Cavity wall temperature (K)	Percentage difference in cavity wall temperature (%)	TPF (–)	Percentage difference in TPF (%)	Effectiveness (%)	Percentage difference in effectiveness (%)
hBN/water	0.1	311.5	0.40	1.51	11.10	7.10	9.67
hBN/water	0.15	312.8	Reference	1.36	Reference	6.48	Reference
hBN/water	0.2	313.7	0.29	1.27	6.69	6.09	5.92
G/water	0.1	310.2	0.51	1.70	15.84	7.61	13.79
G/water	0.15	311.8	Reference	1.47	Reference	6.69	Reference
G/water	0.2	313.0	0.35	1.34	8.58	6.18	7.62
GO/water	0.1	316.4	0.07	1.07	1.27	5.23	1.03
GO/water	0.15	316.6	Reference	1.05	Reference	5.17	Reference
GO/water	0.2	316.8	0.06	1.04	1.13	5.12	1.05

#### A1.2. Sensitivity to platelet viscosity model

The viscosity of platelet-based nanofluids depends on the particle aspect ratio and packing characteristics. To evaluate the influence of viscosity modeling assumptions, a sensitivity analysis was performed using the Krieger–Dougherty viscosity model,^72^ given as follows:A1
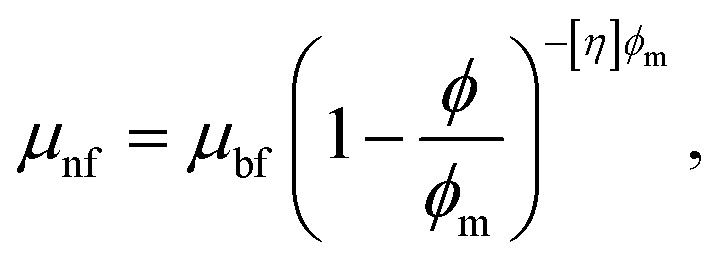
where [*η*] is the intrinsic viscosity and *ϕ*_m_ is the maximum packing fraction. In the present study, the viscosity calculated from eqn (7) corresponds to [*η*] = 6.04 and *ϕ*_m_ = 0.17 in the Krieger–Dougherty viscosity model for *ϕ* = 5%. These values fall within the literature ranges reported for platelet suspensions, including the values proposed by Kasgoz *et al.*^73^ and Claypole *et al.*^74^Table 8 compares the predicted viscosity, cavity wall temperature, thermal performance factor, thermal effectiveness, and pumping power obtained using these parameter sets. The results show that the predicted thermohydraulic behavior is only weakly sensitive to the viscosity model parameters. The nanofluid viscosity varies less than 4.4%, leading to changes below 0.07% in cavity wall temperature, 1.5% in TPF and effectiveness, and 0.5% in pumping power. These small deviations confirm that the main conclusions of the study are not significantly affected by reasonable variations in platelet viscosity modeling.

**Table 8:** Sensitivity of the predicted thermohydraulic performance to platelet viscosity modeling using the Krieger–Dougherty relation at a nanoparticle volume fraction of *ϕ* = 5%. Percentage differences relative to the present study are shown in parentheses

Reference	Intrinsic viscosity [*η*]	Maximum packing fraction (*ϕ*_m_)	Viscosity (Pa s^−1^)	Cavity wall temperature (K)	TPF	Effectiveness (%)	Pumping power (W)
Kasgoz *et al.*^73^	5.4	0.189	0.001217 (4.40%)	311.64 (0.067%)	1.494 (1.79%)	6.77 (1.13%)	0.275 (0.93%)
Present work	6.04	0.17	0.001273 (reference)	311.85 (reference)	1.468 (reference)	6.69 (reference)	0.278 (reference)
Claypole *et al.*^74^	6.37	0.151	0.00131 (2.91%)	311.99 (0.047%)	1.450 (1.19%)	6.63 (0.84%)	0.280 (0.58%)

## Supplementary Material

NA-OLF-D6NA00038J-s001

## Data Availability

The data supporting this article have been included as part of the supplementary information (SI). Supplementary information is available. See DOI: https://doi.org/10.1039/d6na00038j.
